# Expert opinion—management of chronic myeloid leukemia after resistance to second-generation tyrosine kinase inhibitors

**DOI:** 10.1038/s41375-020-0842-9

**Published:** 2020-05-04

**Authors:** Andreas Hochhaus, Massimo Breccia, Giuseppe Saglio, Valentín García-Gutiérrez, Delphine Réa, Jeroen Janssen, Jane Apperley

**Affiliations:** 1 Klinik für Innere Medizin II, Universitätsklinikum Jena, Jena, Germany; 2 Sapienza University of Rome, Rome, Italy; 3 University of Turin, Turin, Italy; 4 Hospital Universitario Ramón y Cajal (IRYCIS), Madrid, Spain; 5 Hôpital St. Louis, Paris, France; 6 Department of Hematology, Cancer Center Amsterdam, Amsterdam University Medical Centers, loc. VUMC, Amsterdam, The Netherlands; 7 Hammersmith Hospital, Imperial College London, London, UK

**Keywords:** Chronic myeloid leukaemia, Clinical trials

## Abstract

Regardless of line of therapy, treatment goals in chronic phase chronic myeloid leukemia (CML) are: avoid progression to accelerated phase or blast crisis CML such that patients achieve a life expectancy comparable with that of the general population; avoid adverse events (AEs); and restore and maintain quality of life. The most important prognostic factor for achieving these goals is response to tyrosine kinase inhibitors (TKIs) at key milestones. For patients failing a TKI, a treatment change is mandatory to limit the risk of progression and death. There is currently no precise guideline for patients that fail a second-generation TKI, and there is a paucity of data to guide clinical decision making in this setting. There is, therefore, an unmet need for practical and actionable guidance on how to manage patients who fail a second-generation TKI. Although the term ‘failure’ includes patients failing for resistance or intolerance, the focus of this paper is failure of a second-generation TKI because of resistance. CML patients who fail their first second-generation TKI for true resistance need a more potent therapy. In these patients, the key issues to consider are the relative appropriateness of early allogeneic hematopoietic stem cell transplantation or the use of a further TKI. Selection of the next line of treatment after second-generation TKI resistance should be individualized and must be based on patient-specific factors including cytogenetics, mutation profile, comorbidities, age, previous history of AEs with prior TKI therapy, and risk profile for AEs on specific TKIs. This expert opinion paper is not in conflict with existing recommendations, but instead represents an evolution of previous notions, based on new data, insights, and clinical experience. We review the treatment options for patients resistant to second-generation TKI therapy and provide our clinical opinions and guidance on key considerations for treatment decision making.

## Introduction

Tyrosine kinase inhibitors (TKIs) have transformed long-term outcomes for patients with chronic phase chronic myeloid leukemia (CP-CML) and life expectancy for these patients is now similar to that of the general population [1]. However, the term ‘chronic’ in CML masks the need for active treatment of a disease where most patients will require life-long TKI therapy. There should be a sense of urgency and timely intervention in the management of patients who fail to achieve recommended milestones [2] to ensure that CP-CML does not progress to a more aggressive disease. There are five TKIs currently approved for the treatment of CP-CML: imatinib, the first-generation TKI; nilotinib, dasatinib, and bosutinib, second-generation TKIs; and ponatinib, a third-generation TKI. The majority of patients with CP-CML receive imatinib as first-line (1L) treatment and achieve good long-term disease control [3]. Increasingly, patients receive a second-generation TKI as 1L treatment [4] for a variety of reasons. These include patient- and disease-related factors, treatment-related factors [5–8], and the goal of higher and/or faster probabilities of a treatment-free remission (TFR) attempt. However, no survival benefit has yet been demonstrated for any frontline second-generation TKI over imatinib [2, 5, 9].

There are some data for outcomes with second-generation TKI after imatinib failure [10–12]. In contrast, there is a paucity of data to guide clinical decision-making following failure of second-generation TKI, whether this has been used in 1L or second-line (2L) therapy. Currently, there is no precise guideline for patients that fail a second-generation TKI: existing recommendations include (at the same level) the use of another second-generation TKI, ponatinib, a clinical trial, or allogeneic hematopoietic stem cell transplantation (allo-HSCT). There is therefore an unmet need for practical and actionable guidance on how to manage patients who fail a second-generation TKI.

## Purpose of this paper and methodology

CML patients who fail their first second-generation TKI for true resistance need a more potent therapy. In these patients, the key issues to consider are the relative appropriateness of early allo-HSCT or the use of a further TKI. Selection of the next line of treatment after second-generation TKI resistance should be individualized and must be based on patient-specific factors including cytogenetics, mutation profile, comorbidities, age, previous history of adverse events (AEs) with prior TKI therapy and risk profile for AEs on specific TKIs. This expert opinion paper is not in conflict with existing recommendations, but instead represents an evolution of previous notions, based on new data, insights, and clinical experience. The panel members review treatment options for patients resistant to second-generation TKI therapy and provide our clinical opinions and guidance on key considerations for treatment decision making. Panel members corresponded via teleconference calls and mail exchanges to finalize an agreed consensus. No honoraria were received for this project.

## Goals of treatment in CP-CML

Regardless of line of therapy, treatment goals in CP-CML are to avoid progression to accelerated phase or blast crisis CML such that patients achieve a life expectancy comparable with that of the general population; to avoid AEs; and to restore and maintain quality of life. For specific categories of patients, TFR can also be considered a potential goal of 1L therapy once appropriate and sustained clinical response has been achieved. Response to TKI is the most important prognostic factor: only patients achieving complete cytogenetic response (CCyR) or major molecular response (MMR) at key milestones achieve good outcomes. For patients failing a TKI, a treatment change is mandatory to limit the risk of progression and death.

## Definition of ‘failure’ to TKI treatment

Although the term ‘failure’ includes patients failing for resistance or intolerance, the focus of these recommendations is failure of a second-generation TKI because of resistance. Primary resistance indicates a failure to achieve a target response at a given time point, while secondary resistance indicates loss of a prior response [13].

There are precise recommendations for monitoring molecular response by regular assessment of *BCR-ABL1* transcript levels at critical milestones (3, 6, and 12 months). Real-time quantitative reverse-transcription polymerase chain reaction should be used and results reported on the International Scale using an appropriate control gene. The European LeukemiaNet (ELN) [2] and European Society for Medical Oncology (ESMO) [14] have defined TKI failure and are generally aligned. ELN 2020 definitions for failure are summarized in Table 1 [2].

**Table 1:** ELN 2020 definitions of failure to 1L and 2L treatment [2].

**Time**	**Definition of TKI failure**
3 months	*BCR-ABL1* (IS) > 10% if confirmed within 1–3 months
6 months	*BCR-ABL1* (IS) > 10%
12 months	*BCR-ABL1* (IS) > 1%
Any time	*BCR-ABL1* (IS) > 1%, resistance mutations, high-risk ACA

*1L* first-line, *2L* second-line, *ACA* additional chromosome abnormalities*, ELN* European LeukemiaNet, *IS* International Scale, *TKI* tyrosine kinase inhibitor.

From a practical perspective, when should a patient be considered as failing a second-generation TKI? ELN 2013 recommendations defined failure to 1L and 2L therapy with different milestones, being less stringent for 2L therapy [15]. The 2020 version has changed this view. The need for a more stringent definition of failure has been implemented, such that those patients not achieving *BCR-ABL1* ≤ 1% (or CCyR) at 12 months, including those receiving 2L therapy, should be considered as failing treatment [2].

## Treatment options after resistance to a second-generation TKI

An important minority of patients becomes resistant to a second-generation TKI and will require an alternative treatment. Note that the situation for patients failing a second-generation TKI in the 1L setting versus those failing a second-generation TKI in the 2L setting differs and the route to resistance is different [16]: patients failing a 1L second-generation TKI may represent a population of patients with an unfavorable prognosis.

**Fig. 1:**
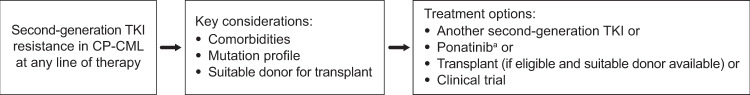
Considerations and treatment options after second-generation TKI resistance. ^a^Ponatinib dose based on comorbidities and mutation profile.

For patients with CP-CML who are resistant to a second-generation TKI, mutational analysis should be performed, comorbidities assessed, and the search for a suitable donor for allo-HSCT should be initiated. Treatment should then be decided based on mutation profile and comorbidities (Fig. 1).

*BCR-ABL1* mutation analysis should be carried out following failure of a second-generation TKI, using either conventional Sanger sequencing or the more sensitive next-generation sequencing (NGS). Mutation results can guide selection of the most appropriate TKI and prevent the use of an inappropriate TKI. Heat maps [17, 18] and tables [7, 19, 20] are available to guide second-generation TKI selection according to mutation type. NGS can detect low-level mutations present below the sensitivity threshold of Sanger sequencing and can reveal compound mutations. However, at the time of documented resistance, these low-level mutations may not be drivers of resistance to TKI therapy and generally do not guide TKI selection. One exception, however, is the detection of the *BCR-ABL1* T315I mutation that would prompt use of ponatinib, even if present at low levels. Although the role of compound mutations as drivers of TKI resistance has not been clearly defined, they are of clinical concern, and their detection would tend to support selection of ponatinib or allo-HSCT.

A key recommendation is that the search for a donor should commence as soon as the patient fails a second-generation TKI: allo-HSCT can offer the prospect of long-term survival for eligible patients. Though a fully human leukocyte antigen (HLA)-matched related donor is optimal for HSCT, approximately two-thirds of patients requiring HSCT do not have a matched related donor and rely on the identification of an HLA-matched unrelated or a haploidentical donor. The search for a matched unrelated donor can take on average 3–4 months, during which time patients may progress or become unfit for transplant [21], and in this situation the use of a haploidentical donor may be the preferred option. For patients who are not transplant eligible and who have exhausted all available TKIs, a trial of an exploratory treatment is appropriate.

Following resistance to a second-generation TKI, an alternative second-generation TKI might be an option (e.g., after resistance to imatinib and nilotinib, subsequent treatment with dasatinib or bosutinib is feasible, also depending on specific mutations, if present). However, treatment with a third-generation TKI (ponatinib) should be considered for all eligible patients. A role for earlier ponatinib use is especially evident for patients demonstrating resistance to second-generation TKIs in both the 1L and 2L settings. Our recommendation is based on evidence from various studies. Less than 10% of patients (*N* = 113) receiving a second-generation TKI (nilotinib or dasatinib) who fail to achieve a cytogenetic response at 3–6 months eventually attained the target of major cytogenetic response (MCyR) at 12 months [22]. Although there are no head-to-head trials, and reported studies enrolled low patient numbers, in the absence of a mutation sensitive to an alternative second-generation TKI, there appears to be limited value of using another second-generation TKI after failure of a prior second-generation TKI. Response rates (CCyR) for sequential nilotinib/dasatinib range from around 10 to 35% across studies in third-line (3L) or later, and many of the patients who responded received the second-generation TKI for intolerance rather than resistance (Table 2). Furthermore, only low numbers of patients remained on treatment, indicating a substantial rate of failure across studies [23–28]. Primary results from the phase 4 BYOND study of bosutinib in second and later-line therapy of CML demonstrated a CCyR rate of 84% in the third-line setting (*n* = 56; Table 2) [29]. When response was assessed according to resistance or intolerance across the whole population (evaluable *n* = 144) and irrespective of line of therapy, CCyR rates were similar (77% and 87% in resistant and intolerant patients, respectively) though MMR rates were lower for resistant patients (46%; *n* = 48) compared with intolerant patients (81%; *n* = 31) [29].

**Table 2:** Response to second-generation TKIs in 3L (or later) treatment of CP-CML^a,b^.

**Study**	**TKI**	**Cumulative CCyR rate, %**	**Cumulative MMR rate, %**	**Median follow-up, months (range)**	**EFS/PFS/TTF**	**OS**	**Prior treatment failure information,** ***n*/*N***	**Mutations in pts tested prior to 3L therapy,** ***n*/*N***
Garg 2009 [23] (*N* = 48 total group) (*n* = 25 CP; *n* = 10 AP; *n* = 13 BC)	Dasatinib 3L in CP-CML (*n* = 16)	31	13	13 (0.5–41) (all pts)	Median FFS: 20 months	Median OS: 20 months (all pts)	30/34 (all pts) failed prior 2L TKI due to resistance	11/16
	Nilotinib 3L in CP-CML (*n* = 9)	11	33				9/14 (all pts) failed prior 2L TKI due to resistance	7/9
Ribeiro 2015 [24] (*n* = 18)	Dasatinib 3L (*n* = 5) or nilotinib 3L (*n* = 13)	13^c^	24^c^	52 (7–75)	5-year EFS: 22%	5-year OS: 86%	16/18 failed prior 2L TKI due to resistance and 2/18 due to intolerance	6/14
					5-year PFS: 54%			
Lomaia 2015 [25] (*n* = 53)	Dasatinib (*n* = 30), nilotinib (*n* = 18), or bosutinib (*n* = 5) 3L	21	NA	21 (1–67)	NA	2-year OS: 67%	48/53 pts failed one TKI 42/53 pts failed both prior TKIs	16/35 (T315I in 3 pts)
Giles 2010 [26] (*n* = 60 total group) (*n* = 39 CP; *n* = 21 AP)	Nilotinib 3L in CP-CML (*n* = 39)	24^d^	NA	12 (NA) (all pts)	Median TTF: 19.5 months	18-month estimated OS: 86%	12/39 failed prior 2L TKI (dasatinib) due to resistance, 26/39 due to intolerance, and 1/39 due to an undefined reason	12/25 Most common: F317L (*n* = 6); T315I (*n* = 2)
					18-month estimated PFS: 59%			
Cortes 2011 [27] (*n* = 29)^e^	Any TKI 3L or later^e^	24^e^	NA	NA for 3L and beyond cohort	NA	NA	NA	NA
Ibrahim 2010 [28] (*n* = 26)	Dasatinib or nilotinib 3L^f^	35	19	21.5 (6–46.5)	30-month EFS: 46%	30-month OS: 47%	7/26 pts had prior hematologic resistance to TKI	NA
Hochhaus 2019 [29] (*n* = 61)	Bosutinib 3L	84 (resistant or intolerant; *n* = 56)^g^	64 (resistant or intolerant; *n* = 28)^h^	NA for 3L cohort	NA for 3L cohort	NA for 3L cohort	35/61 resistant to any prior TKI	NA
							26/61 intolerant to prior TKIs	

*2L* second-line, *3L* third-line, *4L* fourth-line, *AP* advanced phase, *BC* blast crisis, *CML* chronic myeloid leukemia, *CP* chronic phase, *CCyR* complete cytogenetic response, *EFS* event-free survival, *FFS* failure-free survival, *MMR* major molecular response, *NA* not available, *OS* overall survival, *PFS* progression-free survival, *pts* patients, *TKI* tyrosine kinase inhibitor, *TTF* time to treatment failure.

^a^The heterogeneity of the studies should be noted with some being small non-controlled studies and some being larger registrational trials.

^b^Resistant/intolerant CP-CML patients receiving ≥3L TKI are counted (unless specified as ‘all pts’).

^c^CCyR data are based on 15 patients and MMR data are based on 17 patients.

^d^In Giles 2010 study, 39 patients were enrolled and 37 evaluable for response. Response data are based on 37 patients; however, resistance to 2L TKI data are based on the 39 patients enrolled.

^e^In Cortes 2011 study, 26 patients were treated in 3L and a further 4 in 4L (of whom 3 were evaluable). Response data are reported for all 29 evaluable patients in 3L and 4L. Of the 26 3L patients: 15 (58%) had received prior dasatinib; 7 (27%) prior nilotinib; 2 (8%) prior bosutinib; and 2 (8%) prior bafetinib.

^f^In Ibrahim 2010 study, data are reported together for dasatinib-treated or nilotinib-treated patients.

^g^Cumulative rates in patients evaluable for response; CCyR rate was 77% in resistant patients treated in 2/3/4L (*n* = 77).

^h^Cumulative rates in patients evaluable for response, excluding patients with baseline MMR; MMR rate was 46% in resistant patients treated in 2/3/4L (*n* = 48).

In a phase 1/2 study of bosutinib in 3L or later, the probability of newly attained CCyR was 26% and, after 4 years of follow-up, only 24% of patients were still on treatment [30]. A recent retrospective analysis of the largest cohort of patients (*N* = 62) treated with fourth-line bosutinib, after failing imatinib, nilotinib, and dasatinib, reported a 25% probability of achieving or maintaining CCyR and 24% probability of achieving MMR (median 14 months follow-up). However, patients not in CCyR at the time of bosutinib start were least likely to achieve a molecular response (14% probability of achieving MMR) [31].

The PACE trial was a phase 2 study of ponatinib in patients with Philadelphia chromosome-positive CML or acute lymphoblastic leukemia resistant/intolerant to dasatinib or nilotinib, or carrying the *BCR-ABL1* T315I mutation. Final 5-year results demonstrated that 54% and 60% of CP-CML patients resistant to two or more prior TKIs achieved CCyR and MCyR, respectively, at any point, with 82% of responders estimated to remain in MCyR at 5 years [32]. CP-CML patients who received fewer prior TKIs attained higher cytogenetic and molecular responses. Of CP-CML patients previously treated with one (*n* = 16), two (*n* = 98), three (*n* = 141), or four (*n* = 12) prior TKIs, 75%, 70%, 49%, and 58% achieved an MCyR, respectively; 63%, 42%, 36%, and 8% achieved an MMR, respectively [33]. The median time to MCyR or CCyR was within 3 months [32], which would allow early identification of patients unlikely to respond and aligns with the period for donor search.

The starting dose of ponatinib for patients with CP-CML (15, 30, or 45 mg/day) should be decided based on comorbidities and mutation profile (e.g., being aware that a higher dose may be required for patients with aggressive mutations such as E255V or compound mutations). Dose adjustments should then be made according to response and tolerability. However, dose reduction may lead to loss of response. In the PACE trial, preemptive dose reductions were implemented to decrease the risk of arterial occlusive events. Overall ≥90% of CP-CML patients who had achieved MCyR or MMR maintained response 40 months after elective dose reductions [32]. A retrospective analysis of low-dose (15 mg) ponatinib as a starting or de-escalated dose in CP-CML patients (*N* = 62) reported a 55% CCyR rate, and a response of MMR or better was maintained in 35/54 patients (65%) at a median 21 months follow-up [34]. Registry US data for CP-CML patients receiving ponatinib (*n* = 475) indicate that 47% received a starting dose of 45 mg/day, 29% received 30 mg/day, and 24% received 15 mg/day [35]. The mutation status should also be considered when considering dose adjustments, as the concentration should be adequate to suppress mutations and provide disease control. Prospective dose evaluation studies are ongoing (e.g., OPTIC, NCT02467270) [36–38] and may provide information on the optimal starting dose for ponatinib.

Each TKI has an associated toxicity profile, which requires certain patients to be carefully selected and monitored during treatment (Table 3). Factors that need to be considered before selecting ponatinib include patients’ cardiovascular (CV) risk, metabolic disease, concomitant medications, and comorbidities. Although CV AEs have been reported for all TKIs, the relative risk is highest with ponatinib [32, 39]. Thus, the potential benefits of ponatinib treatment must be balanced against the potential risks, but there is no absolute contraindication of any TKI based on comorbidities.

**Table 3:** Cardiovascular, pulmonary, and metabolic AEs associated with each TKI (Adapted from [40]).

**TKI**	**Associated CV, pulmonary and metabolic AEs**	**Patients who require careful monitoring and caution advised**
Imatinib	Congestive heart failure and left ventricular dysfunction	Patients with cardiac disease
	Rare pulmonary toxicity	Patients with risk factors for cardiac failure
Dasatinib	Pulmonary arterial hypertension, pleural effusions, pneumonitis	Patients with preexisting cardiopulmonary disease
	QT prolongation	Patients who may develop QT prolongation
Nilotinib	QT prolongation	Patients at risk for hyperlipidemia or hyperglycemia
	Cardiac and arterial vascular occlusive events	Avoid in patients with long QT syndrome
	Hyperlipidemia or hyperglycemia	Avoid in patients with hypokalemia or hypomagnesemia
	Sudden deaths have been reported in CP patients with imatinib-resistant/intolerant CML with a history of cardiac disease or significant cardiac risk factors	
	Rare pleural effusions	
Bosutinib	Cardiovascular, pulmonary, and metabolic toxicities are infrequent	Patients with CV risk factors
	Rare pleural effusions	
Ponatinib	Vascular occlusion	Patients with hypertension
	Heart failure	Patients at risk for arrhythmias
	Hypertension	Patients at risk for heart failure
	Arrhythmias	Patients with preexisting cardiopulmonary disease
	Possible pulmonary hypertension	

*AE* adverse event, *CML* chronic myeloid leukemia, *CP* chronic phase, *CV* cardiovascular, *TKI* tyrosine kinase inhibitor.

## Treatment options for patients in whom ponatinib is not appropriate

The clinical picture is complex for patients resistant to a second-generation TKI, but for whom ponatinib is not deemed appropriate. Clear-cut recommendations are not possible for this heterogeneous population who are also likely to be ineligible for transplant. However, another second-generation TKI (depending on mutation profile, comorbidities, previous AEs to TKI therapy, and other factors previously described) or a clinical trial are both rational options.

## Consideration of when transplantation may be appropriate

Early consideration of allo-HSCT is crucial and should be discussed with the patient as soon as possible following second-generation TKI resistance. The heterogeneity of transplant risks (e.g., non-relapse mortality or graft-versus-host disease) means that the decision of whether or not to transplant patients in CP is complex. There is currently no definitive consensus, rather each decision must be based on individual benefit–risk assessment. However, to delay transplant until all available TKIs have been exhausted would be inappropriate for some patients, especially those with unfavorable parameters who may not benefit from further TKI treatment. The presence of high risk additional chromosomal aberrations (complex karyotypes, isochromosome 17, abnormalities in chromosome 3, monosomy 7, and trisomy 8) is a trigger for transplant [41]. In cases without any unfavorable parameters, alternative TKI therapy (or a clinical trial) may be appropriate. Treatment strategies that result in delayed referral for transplant with the associated risk of disease progression that may compromise patient eligibility for allo-HSCT are not recommended. For example, an indication where it may be appropriate to transplant before using ponatinib is in very young patients with an available matched sibling donor. Specific recommendations regarding induction, conditioning, and maintenance regimens are outside the scope of this paper.

## Considered for long-term TKI treatment

The focus of these recommendations is on patients failing second-generation TKI for resistance rather than intolerance. However, for a patient who needs ponatinib but has CV issues (such as a history of myocardial infarction) it may be appropriate to exercise caution and start with a lower dose of ponatinib (if not eligible for allo-HSCT), while for other patients with no CV risk but with an aggressive CML then the ponatinib 45 mg starting dose should be considered. Note that ponatinib dose can be reduced once the desired response is achieved. The approach to patient management must take into account not just AEs but also comorbidities, which (though independent of CML) have an equal impact on treatment choice. Selection of the best treatment option must be personalized to the individual patient, achieving efficacy while preventing AEs. If TKI-related AEs do occur, there is guidance on their management [42].

## TFR after resistance to a second-generation TKI

Although TFR is becoming an increasingly desired goal of treatment, any TFR attempt in patients who have demonstrated resistance to a second-generation TKI would be premature and is not currently recommended.

## Final thoughts: managing patients after second-generation TKI resistance

Despite the range of options discussed, there may be patients for whom none is appropriate (i.e., patients unable to receive ponatinib or a second-generation TKI, and ineligible for clinical trial or allo-HSCT). In such cases, use of interferon-alpha and/or best supportive care for disease and symptom control could be appropriate and realistic treatment options. The CML treatment landscape evolves rapidly with new insights and better understanding driving development of novel therapeutic approaches. Several approaches and hypotheses are being explored, including strategies to overcome *BCR-ABL1*-independent inhibition and mutation-mediated resistance, and strategies targeting leukemia stem cells. For now, these approaches and hypotheses remain exploratory and there are insufficient data or evidence to guide clinical decisions.

Treatment goals for patients in CP-CML with resistance to a second-generation TKI are unchanged regardless of line of therapy. The current evidence base does allow certain recommendations to be made following second-generation TKI resistance. Transplant should be considered early to allow timely initiation of donor search. Eligible patients should receive a third-generation TKI (especially in cases where no known mutation is driving resistance) with dose modification considered as clinically appropriate. Depending on mutation profile, another second-generation TKI may also be feasible. For patients unable to receive ponatinib, clinical trials of newer agents or allo-HSCT if all possible TKI options are exhausted, are options. Asciminib, a TKI that has shown promising phase 1 data in heavily pretreated patients may be a future option for these patients [43].
